# Anti-inflammatory activity of *Jefea gnaphalioides* (a. gray), Astereaceae

**DOI:** 10.1186/s12906-019-2654-x

**Published:** 2019-09-23

**Authors:** Axel Villagómez-Rodríguez, Julia Pérez-Ramos, Ana Laura Esquivel-Campos, Cuauhtémoc Pérez-González, Claudia Angélica Soto-Peredo, Salud Pérez-Gutiérrez

**Affiliations:** Departamento de Sistemas Biológicos, Universidad Autónoma Metropolitana-Xochimilco, Ciudad de Mexico, México

**Keywords:** Anti-inflammatory effect, *Jefea gnaphalioides*, Methanol extract, Composition, Inflammatory interleukin

## Abstract

**Background:**

Inflammation is a symptom associated with many diseases. This symptom is treated with steroidal and non-steroidal anti-inflammatory drugs, which can cause severe side effects when used as long-term treatments. Natural products are an alternative source of new compounds with anti-inflammatory activity. *Jefea gnaphalioides* (Astereaceae) (A. Gray) is a plant species used to treat inflammatory problems, in Mexico. This study determined the anti-inflammatory activity and the composition of the methanol extract of *Jefea gnaphalioides* (MEJG)*.*

**Methods:**

The extract was obtained by heating the plant in methanol at boiling point for 4 h, and then the solvent was evaporated under vacuum (MEJG). The derivatization of the extract was performed using Bis-(trimethylsilyl) trifluoroacetamide, and the composition was determined by GC-MS.

Total Phenols and flavonoids were determined by Folin-Ciocalteu AlCl_3_ reaction respectively.

The antioxidant activity of MEJG was determined by DPPH method.

The acute and chronic anti-inflammatory effects were evaluated on a mouse ear edema induced with 12-O-Tetradecanoylphorbol-13-acetate (TPA).

Acute oral toxicity was tested in mice at doses of MEJG of 5000, 2500 and 1250 mg/kg.

The levels of NO, TNF-α, IL-1β and IL-6 were determinate in J774A.1 macrophages stimulated by Lipopolysaccharide. The production of inflammatory interleukins was measured using commercial kits, and nitric oxide was measured by the Griess reaction.

**Results:**

The anti-inflammatory activity of MEJG in acute TPA-induced ear edema was 80.7 ± 2.8%. This result was similar to the value obtained with indomethacin (IND) at the same dose (74.3 ± 2.8%). In chronic TPA-induced edema at doses of 200 mg/kg, the inhibition was 45.7%, which was similar to that obtained with IND (47.4%). MEJG have not toxic effects even at a dose of 5000 mg/kg. MEJG at 25, 50, 100 and 200 μg/mL decreased NO, TNF-α, IL-1β and IL-6 production in macrophages stimulated with LPS.

The major compounds in MEJG were α-D-Glucopyranose (6.71%), Palmitic acid (5.59%), D-(+)-Trehalose (11.91%), Quininic acid (4.29%) and Aucubin (1.17%). Total phenolic content was 57.01 mg GAE/g and total flavonoid content was 35.26 mg QE/g MEJG had antioxidant activity.

**Conclusions:**

MEJG has anti-inflammatory activity.

## Background

Inflammation is a complex natural response to harmful stimuli, such as physical trauma, noxious chemicals, bacteria and viruses. Inflammation is a defense reaction caused by an injury, and it is seen as a response to many diseases such as cancer, rheumatoid arthritis and diabetes [1–3]. In the injured tissue, activation of immune cells such as macrophages, monocytes, and neutrophils, and production of pro-inflammatory cytokines such as interleukins IL-1β, IL-6 and tumor necrosis factor (TNF)-α are observed. These responses are related to the development and progression of inflammation and autoimmune diseases [4]; IL-10 is the most important anti-inflammatory cytokine, and it is produced by activated immune cells and diminishes the production of inflammatory mediators [5]. Drugs used in the treatment of inflammation are non-steroidal anti-inflammatory drugs (NSAIDs) and steroidal drugs, however, both can cause severe adverse side effects. Thus, the development of new, safe anti-inflammatory compounds is necessary. Plants used in traditional medicine for treating various inflammatory conditions are a source of biologically active compounds, and these plants have been used for a long time as crude material or pure compounds for the treatment of several diseases [6].

*Jefea gnaphalioides* (Astereaceae) (A. Gray) Strother (Basionym: *Zexmenia gnaphalioides*) [7], commonly known as Peonia, is a small shrub with yellow flowers that is native to Mexico and the southwestern United States of America. This plant is used in Mexico in folk medicine for the treatment of diabetes [8] and inflammatory conditions. Valerenal and 10-Epi-β-valerenol have been isolated from *J. gnaphalioides* [9].

We reported previously that the methanol extract from the leaves of this plant had anti-inflammatory activity on ear edema induced by TPA [10]. Based the results of that study, the objective of the present research is to determine the composition of MEJG, and evaluate the in vitro anti-inflammatory effect of MEJG on the production of NO, TNF-α, IL-1β, and IL-6 in J774A.1 macrophages stimulated by Lipopolysaccharide *Escherichia coli* O111:B4 (LPS).

## Methods

### Plant material

Aerial parts of *J. gnaphaloides* were collected in August 2014 in Las Candelas Municipality of Guadalcazar, San Luis Potosí State, Mexico. The material was identified by taxonomist José García Pérez. A voucher specimen (SPLM039513) was deposited in the Isidro Palacios Herbarium of the Universidad Autónoma de San Luís Potosí.

### Preparation of the extract

The dried ground plant (330 g) was defatted in 2.0 L of hexane under reflux for 4 h, and then the material was extracted with methanol (2.0 L) at boiling point for 4 h. The solvent was removed under reduced pressure, and a dark-brown solid was obtained (6.1% yield). The solid was used for the further evaluations. The datasets analyzed during the current study are available from the corresponding author on reasonable request.

### Total phenolic assay

The content of total phenolic of MEJG was determined by Folin-Ciocalteu assay [11] A solution of 100 μL of MEJG (0.1 mg/mL), 800 μL of deionized water and 100 μL of Folin-Ciocalteu reagent for 8 min in dark, followed by the addition of 50 μL of 7.5% (w/v) Na_2_CO_3._ The mixture was to stand at room temperature for 60 min in dark and the absorbance of the mixture was registered at 760 nm. The calibration curve was obtained with gallic acid (0–200 μg/mL). The total phenolic content was expressed as mg of gallic acid equivalents (GAE)/g dry weight. The tests were performed in triplicate.

### Total flavonoids assay

The total flavonoids content of MEJG was determined by de AlCl_3_ colorimetric method [12]. Five hundred microliter of MEJG (I mg/mL ethanol) were added with an equal volume of 2% AlCl_3_ in ethanol. The absorbance of the mixture was obtained at 420 nm. The total flavonoid content was calculated from a quercetin calibration curve (10–60 μg/mL), and the result was expressed as mg quercetin equivalents (QE)/ g dry weight. The assay was performed in triplicate.

### DPPH assay

In assay was using the method of Chanda et al. [13] with modifications. A mixture of 100 μl of 0.208 mM DPPH, and 100 μl of MEJG, dissolved in methanol (1–200 μg/mL). Absorbance was measured at 490 nm after 20 min in the dark. For calculation, a butyl hydroxytoluene (BHT) calibration curve was plotted (1–200 μg/mL).

DPPH scavenging capacity of samples was calculated by the equation:

$$ \%\mathrm{Radical}\ \mathrm{scavenging}\ \left(\mathrm{RSA}\right)=\left(\frac{Ac- As}{Ac}\right)\times \kern0.37em 100 $$

Where Ac = absorbance of the control, and As = absorbance of the samples. Methanol was used as the blank. The assays were performed by triplicate. IC_50_ value is the concentration of the sample required to scavenging 50% of the free radicals.

### Derivatization

Isooctane (1 mL, J.T. Baker) and 100 μL of Bis-(trimethylsilyl) trifluoroacetamide (Sigma-Aldrich) were added to 10 mg of MEJG, and the mixture was heated at 100 °C for 10 min at 150 watts in a microwave oven.

### MEJG analysis

MEJG was analyzed on a gas chromatograph coupled to a mass spectrometer (Agilent Technology, model 7890B) with an HP-5MS capillary column (30 m in length, 0.25 mm internal diameter and 0.25 μm film thickness). A gradient temperature program was used. The initial temperature was 100 °C, and that was held for 3 min. The temperature was then increased up to 320 °C at a rate of 10 °C/min; this temperature was maintained for 10 min. The injector temperature was 320 °C, and the splitless was performed at a ratio of 1:100, and the. The spectrum was determined at 70 eV. The identification of the compounds was determined by a comparison of their experimental mass spectra to those of standard samples and the NIST library (Wiley09/NIST14).

### Experimental animals

Male CD1 mice (20–25 g, 7–8 weeks of age) obtained from the Universidad Autónoma Metropolitana-Xochimilco (UAM-X) animal facility were kept in isolated cages; mice were maintained with food (Lab Diet 5001) and water ad libitum. They were housed in groups at 24 °C ± 1 °C under 12 h light-dark cycles. All experimental protocols were approved by the Research Bioethics Committee of the UAM-X with number 140. All animals were cared for and treated humanely according with the current procedure for the care of animals by the official Mexican Norm (NOM-062-ZOO-1999). The animals were acclimatized to laboratory conditions for 1 week prior to the experiments and they were done after 9:00 am. They were sacrificed in a CO_2_ chamber.

### Evaluation of acute anti-inflammatory activity on TPA-induced mouse ear edema

The method used in this study was described previously [14]. TPA (2.5 μg, Sigma-Aldrich) was dissolved in 25 μL of acetone and the solution was applied topically to the inner and outer surfaces of the right ears of the mice, and 25 μL of acetone was applied to both surfaces of the left ear. Thirty minutes later, MEJG (2.0 mg/ear) or IND (Sigma-Aldrich) dissolved in acetone were topically applied to the right ear. The animals were sacrificed after 6 h, and 6 mm plugs of the central portion of both ears of each mouse were weighed. The percent inhibition of the edema was determined.

$$ \%\mathrm{Inhibition}=\left(\frac{\left({W}_t-{W}_{nt}\right)\mathrm{control}-\left({W}_t-{W}_{nt}\right)\mathrm{treated}}{\left({W}_t-{W}_{nt}\right)\mathrm{control}}\right)100 $$

*W*_*t*_: weight of treated ear, *W*_*nt*_*:* weight of non-treated ear.

### Evaluation of chronic anti-inflammatory activity on TPA-induced mouse ear edema

Groups of 8 mice were used. They were orally administered the following test samples: vehicle (Polyvinylpyrrolidone, Sigma-Aldrich) (PVP), in a 1:4 ratio, 5 mg/kg of IND or 200, 100, 50, 25, 12.5 mg/kg of MEJG. Thirty minutes after the dose was administered, a solution of 2.5 μg of TPA dissolved in 25 μL of acetone was administered topically to the right ear on both the inner and outer surfaces. The treatments and TPA were applied on days 1, 3, 5, 7 and 9 of the experiment. On the final day, the animals were sacrificed 6 h after the last administration, and 6 mm plugs of the central portion of both ears of each mouse were weighed. The percent of inhibition of the edema was determined with the same formula as was used to determine the acute anti-inflammatory activity.

### Evaluation of acute oral toxicity

The OECD Guideline for testing chemicals [15] methodology was followed. MEJG in PVP was prepared in a 1:4 ratio. Groups of 5 mice were used; the groups were the negative control group and the test groups. Doses of MEJG of 5000, 2500 and 1250 mg/kg were administered. The animals were sacrificed 72 h after administration of the dose, and biopsies were performed to identify possible signs of toxicity.

### Cell viability assay

Cell viability was assessed using a modified 3-(4,5-Dimethylthiazol-2-yl)-2,5-diphenyl-2H-tetrazolium bromide (MTT) (Sigma-Aldrich) assay [16].

J774A.1 macrophages (Senna S.A. de C.V.) (8 × 10^4^ cells/well) were seeded in Dulbecco’s Modified Eagle’s Medium (DMEM) (Gibco Co.) in a 96 well plate and treated with MEJG at a concentration of 1 to 200 μg/mL dissolved in DMSO (Sigma-Aldrich). 48 h after of treatment, 10 μL of MTT solution (5 mg/mL in phosphate-buffered saline) was added to each well, and the plate was incubated at 37 °C for other 4 h. Then, the medium was removed, and the formazan crystals were dissolved in DMSO (100 μL). The optical density (OD) was obtained at 540 nm using an ELISA plate reader from BioRad. Six replicate wells were used to determine the viability using the following equation

$$ \%\mathrm{Viability}=\frac{{\mathrm{OD}}_{\mathrm{treated}\ \mathrm{cells}}}{{\mathrm{OD}}_{\mathrm{control}\ \mathrm{cells}}}\times 100 $$

### Determination of the levels of inflammatory interleukins

A commercially available ELISA kit was using to measure the serum levels of IL-1β, IL-6 and TNF-α, according to the manufacturer’s instructions. A microplate reader at 405 nm with a wavelength correction set to 650 nm was using to determine OD. The J774A.1 macrophages were grown to a density of 1 × 10^6^, and then the cells were treated with MEJG at concentrations of 25, 50, 100 or 200 μg/mL, or IND at a concentration of 8.95 μg/mL (25 μM). All samples were incubated for 2 h. After 5 μg/mL of LPS (Sigma-Aldrich) were added and the cells were incubated for an additional 24 h, the cell-free supernatants were collected and stored at − 20 °C until they were analyzed by immunoassays for the quantification of cytokines. The concentrations of IL-1β, IL-6 and TNF-α in the supernatants of the cultures of the J774A.1 cells were determined using a commercial immunoenzymatic test kit (PeproTech).

### Determination of nitric oxide production

The production of nitrite was measured by the Griess reaction [17]. The J774A.1 macrophages (1 × 10^6^ cells/well) were treated with MEJG alone or extract with LPS (5 μg/mL) at MEJG concentrations of 25, 50, 100 and 200 μg/mL. The cells were incubated for 2 h after which LPS was added followed by an additional 24 h of incubation. The supernatant was collected, and 100 μL were treated with 100 μL of the Griess reagent (Sigma-Aldrich). The mixture was incubated at 37 °C for 15 min, and then the absorbance was measured at 540 nm with a microplate reader.

### Statistical analysis

The results are expressed as the means ± SD. Statistical analyses were performed using Student’s *t*-test and ANOVA followed by Tukey’s test. The statistical significance was set to *p* < 0.05.

## Results

### Composition of MEJG

**Fig. 1:**
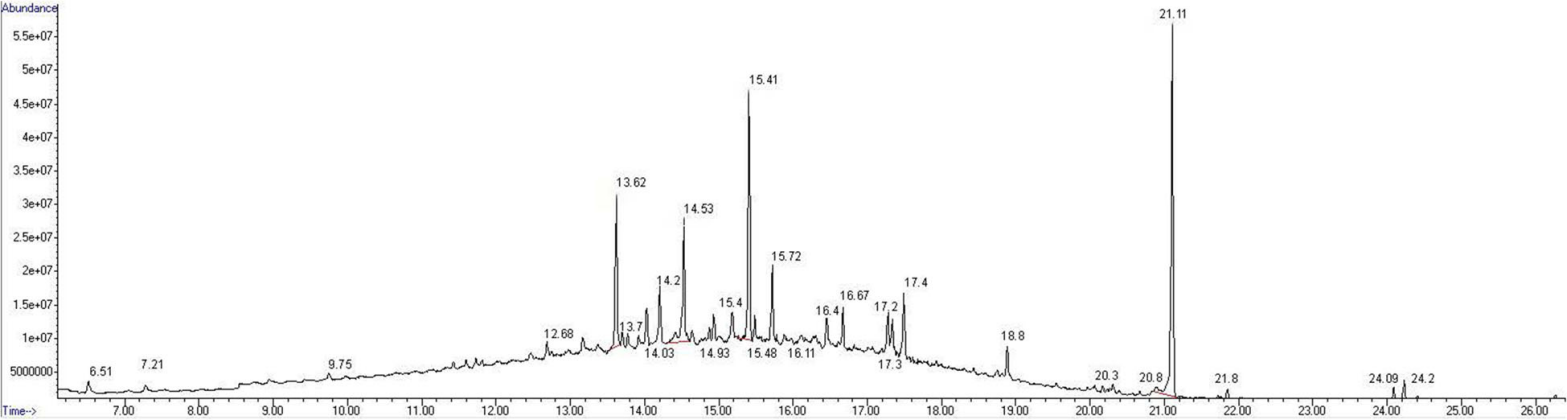
Chromatogram of MEJG

A total of 31 compounds were identified by GC/MS in MEJG derivatizated by silylation, which is one of the most suitable method to obtain derivatives of all compounds and is applied for compounds no volatile, with nitrogen or oxygen with interchangeable hydrogen [18, 19]. The chromatogram of MEJG is shown in Fig. 1. These 31 compounds represent 91.96% of the extract (Table 1), and their retention times are between 6.51 to 24.23 min. The main compounds were D-(+)-Trehalose (11.91%), β-D-Glucopyranose (9.46%), α-D-Glucopyranose (6.71%), Palmitic acid (5.59%), Quininic acid (4.29%), Caffeic acid (3.21%) and Aucubin (1.17%).

**Table 1:** GC-MS Analysis of the Composition of MEJG

Number	Compound	Retention time (min)	%	KI Value
1	Glycerol	6.51	0.61	1108
2	2,3-Dihydroxy-2-methylpropanoic acid	7.28	0.36	1278
3	2,2-Dimethylhexane,	9.75	0.35	732
4	Xylitol	12.68	0.54	1746
5	L-(−)-Sorbofuranose	13.62	5.36	2029
6	β-D-Galactofuranoside	13.7	0.91	1981
7	D-(+)-Talofuranose	14.03	1.99	1991
8	Quininic acid	14.2	4.29	2152
9	α-D-Glucopyranose	14.53	6.71	2037
10	Galactopyranose	14.64	2.18	2037
11	2-Propylpentanoic acid,	14.87	1.77	2651
12	D-Mannitol	14.93	2.03	2066
13	Scyllo-Inositol	15.18	3.54	2194
14	β-D-Glucopyranose	15.41	9.46	2037
15	Myo-Inositol	15.48	2.07	2137
16	Palmitic Acid	15.72	5.59	1987
17	2,2,4-Trimethyl-1-pentanol	15.88	2.83	911
18	2,2-Dimethyl-1-hexanol	16.11	3.22	975
19	Butyl isobutyl oxalic ester	16.3	4.03	1286
20	Muco-Inositol	16.45	4.89	2194
21	Caffeic acid	16.67	3.21	1972
22	(E)-9-Octadecenoic acid	17.28	2.82	2194
23	(Z)-Oleic Acid,	17.33	3.63	2194
24	Stearic acid	17.49	3.71	2186
25	Callitrisic acid	18.88	1.18	2380
26	α-D-Glucopyranosiduronic acid, 3-(5-ethylhexahydro-2,4,6-trioxo-5-pyrimidinyl)-1,1-dimethylpropyl	20.31	0.27	3554
27	Aucubin	20.89	1.17	3323
28	D-(+)-Trehalose,	21.11	11.91	3560
29	D-(+)-Turanose,	21.85	0.28	3572
30	5-O-Feruloylquinic acid	24.09	0.41	3501
31	Chlorogenic acid	24.23	0.66	3610

### The antioxidant activity, and Total phenolic and flavonoid content

The antioxidant activity of MEJG was determined by DPPH assay. This extract had radical scavenger activity against the radicals with IC_50_ value of 31.43 ± 2.69 μg/mL, and the positive control (BHT) presented IC_50_ of 12.42 ± 0.355 μg/mL. The results showed that total phenolic content in MEGJ was 22.04 mg GAE/g dry weight and total flavonoid was 11.63 mg QE/g dry weight.

### Anti-inflammatory activity in vivo

The anti-inflammatory activity of MEJG on acute TPA-induced ear edema in mice was tested at doses of 2 mg/ear. This extract diminished the edema by 80.7 ± 2.8%, which is similar to the inhibition observed with IND at the same dose (74.3 ± 2.8%).

MEJG significantly reduced chronic edema induced by TPA (Table 2). Doses of 12.5 mg/kg did not show any effect in this model; however, at doses of 50 mg/kg (33.2%) and 200 mg/kg (45.7%), the anti-inflammatory effect is similar to that obtained with the positive control, 5 mg/kg of IND, (47.7%). MEJG also shows anti-inflammatory activity in 10-day assays.

**Table 2:** Anti-inflammatory activity of MEJG in chronic TPA-induced edema

% of inhibition	IND	MEJG		
	5 mg/kg	200 mg/kg	50 mg/kg	12.5 mg/ kg
	47.4 ± 3.4	45.7 ± 5.5	33.2 ± 3.0^**^	17.5 ± 3.0^**^

The data were expressed as means ± SD. **p < 0.01, IND vs 200, 50, 12.5 mg/Kg. (*n* = 8)

### Acute oral toxicity of MEJG

No changes in the behavior of the mice were observed during the 72 h after administration of MEJG, and no damage to the liver, heart, intestine, lung and kidney of mice were observed in the biopsies even at doses of 5000 mg/kg.

### Cell viability in macrophages

**Fig. 2:**
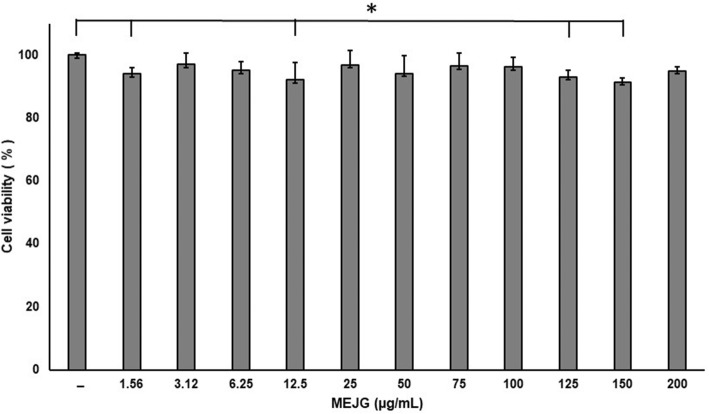
Cell viability in macrophages. Cells were treated with MEJG at 1.56, 3.12, 6.25, 12.5, 25, 50, 100, and 200 μg/ml, and the viability was determined by MTT assays. The results are expressed as the percentage of surviving cells relative to control cells. The data were obtained from three independent experiments and are expressed as the means ± SD *p < 0.5 (*n* = 8)

The cell viability of the J774A.1 macrophages showed that MEJG at concentrations of 1.56 to 200 μg/mL did not affect cell viability (Fig. 2). This extract did not demonstrate any toxicity at the concentrations tested. Therefore, we used 25, 50, 100 and 200 μg/mL of MEJG in subsequent experiments.

### Nitric oxide and inflammatory interleukins levels

**Fig. 3:**
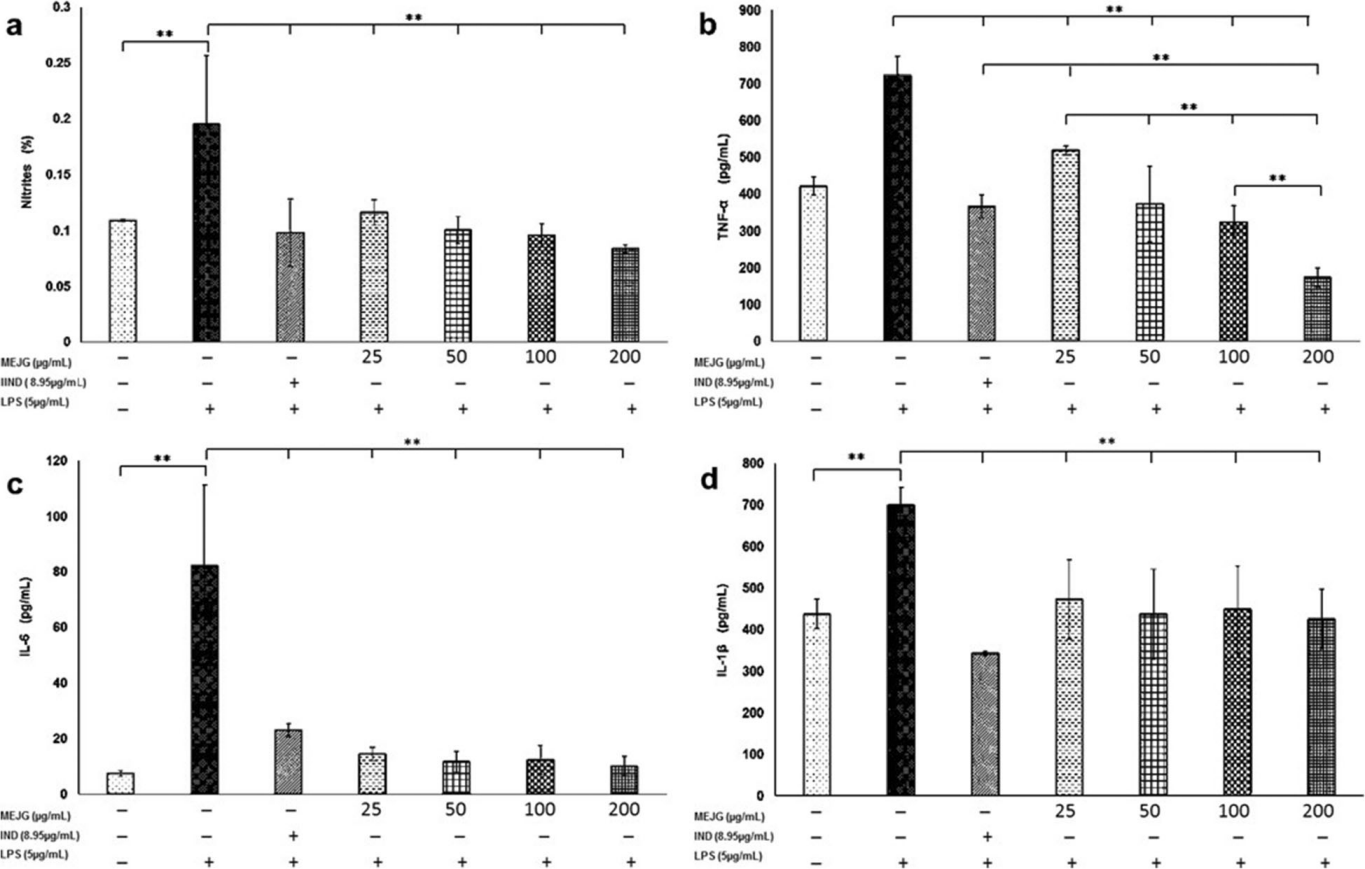
Effects of MEJG on J774A.1 macrophages stimulated with LPS. **a** Nitrites, **b** TNF-α, **c** IL-6 and (**d**) IL-1β. The data were obtained from three independent experiments and are expressed as the means ± SD ** *p <* 0.01 (n = 8)

In this study, was found that MEJG at 25, 50, 100 and 200 μg/mL (Fig. 3a) decreased NO production in macrophages stimulated with LPS, and the effect was independent of concentration. The inhibition of NO production with MEJG was similar to that observed for IND (8.95 μg/mL).

Macrophages stimulated with LPS increase the production of TNF-α, IL-1β and IL-6 inflammatory interleukins (Fig. 3b-d). The cells treated with MEJG showed decreased production of TNF-α at 25, 50, 100 and 200 μg/mL (Fig. 2b); the effect was dependent of concentration. Only doses of 50 and 100 μg/mL showed similar activity to what was observed with IND. At a concentration of 200 μg/mL, TNF-α levels were lower than those obtained with IND.

Additionally, MEJG decreased the levels of IL-6 and IL-1β. The effect was independent of the concentration (25, 50, 100 and 200 μg/mL), but it was similar to that obtained with IND (Fig. 2c-d).

## Discussion

Oxidative processes in humans are responsible for the formation of various species of activated oxygen (ROS), which are involve in the pathology of various human diseases, including atherosclerosis, inflammation, cancer, between others. Plants could be a source of compounds with antioxidant activity [20], and this activity are mainly attributed to phenolic compounds [21] like caffeic acid, 5-O-Feruloylquinic acid, chlorogenic acid, and flavonoids [22] and in case of of MEJG.

Flavonoids inhibit reactive oxygen formation and protect antioxidant defenses [23]. MEJG can to scavenge free radicals, this effect could be due to the phenolic and flavonoids present in the extract and this activity could be one of the mechanisms of MEJG anti-inflammatory activity, because one of the inflammatory responses is the oxidative burst that occurs in different cells [24].

The inflammation produced by topical TPA application is mediated by protein kinase C, the stimulation of phospholipase A_2_, and cyclooxygenase [25]. The inhibition of phospholipase A_2_ is one of the ways to control the inflammation. [26].

The extract significantly diminished acute TPA-induced ear edema in mice which might also suggest that, this extract may be inhibited phospholipase A_2_ and cyclooxygenase.

Additionally, the anti-inflammatory activity of MEJG at different doses was tested on chronic TPA-induced edema (TPA applied 5 times in 10 days). This pharmacological model cause infiltration of inflammatory cells (polymorphonuclear leukocytes) and epidermal hyperplasia [27]; this increases the size of the ear edema. We found that MEJG significantly diminishes the chronic ear edema induced by TPA, and these results suggest that the extract decreased the cellular infiltration.

MEJG presents very low toxicity even at the highest dose (5000 mg/kg). These results suggest the possibility of using the extract in the treatment of inflammatory diseases, but its application requires further study.

Additionally, the cell viability assay showed that the cytotoxicity of this extract was very low even at the highest concentration (200 μg/mL).

Macrophages treated with LPS exhibit a response to this endotoxin, which induce the production of several inflammatory modulators such as NO, TNF-α, IL-6 and IL-1β. NO plays an important role in the pathogenesis of inflammation, and the production of this compound can be used as a measure of the progression of inflammation [28]. Therefore, NO inhibitors are important therapeutic targets in the treatment of inflammatory diseases.

TNF-α, also known as cachectin, is produced by macrophages in response to immunological stimuli such as LPS, and this cytokine is a central regulator of inflammation. It regulates other pro-inflammatory cytokines such as IL-6, which is responsible for the induction and perpetuation of inflammation [29]. Both cytokines can produce severe tissue damage, and their inhibition can be an effective method for treating inflammatory diseases such as rheumatoid arthritis, psoriasis, and ankylosing spondylitis [30].

IL-1β is also a potent pro-inflammatory cytokine that plays an important role in host-defense responses to infection injury [31], and this protein is expressed in many cells including macrophages. This mediator increases the damage during chronic diseases and acute tissue injury [32]. Thus, inhibitors of the production of IL-1β may be effective in the treatment of inflammatory disorders.

Some researches have shown that Aucubin inhibits the production of TNF-α, IL-6 [33] and IL-1β; Wang reported that Aucubin prevents IL-1β-induced inflammation in rat chondrocytes [34], also, it was found that this compound inhibited TNF-α induced secretion and mRNA synthesis of the atherogenic adipokines including PAI-1, MCP-1 and IL.6 [35].

Caffeic acid was found in MEJG (3.21%) prevent damage induced by IL-1β in culture of in vivo organ of articular cartilage [36].

In our study was found that MEJG has anti-inflammatory activity and diminishes the production of NO, TNF-α, IL-1β and IL-6. Several researches have reported that aucubin [33, 34], phenols as caffeic acid [36], and flavonoids components exhibited anti-inflammatory activity in several inflammatory models; therefore, the results suggest that these natural products which exist in MEJG could be responsible for the anti-inflammatory activity of this extract, and for the inhibition of the production of these mediators by acting on NF-кB, which regulates the release of these cytokines. Further studies must be performed to confirm this suggestion.

## Conclusion

MEJG has important anti-inflammatory activity on acute and chronic TPA-induced models. The extract inhibited the release of inflammatory mediators such as NO, TNF-α, IL-6 and IL-1β in macrophages stimulated with LPS. Caffeic acid and Aucubin might be the compounds responsible for the anti-inflammatory activity. MEJG shows low toxicity in mice. These results suggest the possibility of using MEJG for the treatment of inflammation.

## Data Availability

The datasets analyzed during the current study are available from the corresponding author on reasonable request.

## Abbreviations

DMEM: Dulbecco’s Modified Eagle’s Medium
DPPH: 2,2-di phenyl − 1-picrilhidrazil
IL-1β: Interleukin-1β
IL-6: Interleukin-6
IND: Indomethacin
LPS: Lipopolysaccharide *Escherichia coli* O111:B4
MEJG: Methanol extract of the aerial parts of *Jefea gnaphalioides*
MTT: 3-(4,5-Dimethylthiazol-2-yl)-2,5-diphenyl-2H-tetrazolium bromide
NO: Nitric Oxide
NSAIDs: Non-steroidal anti-inflammatory drugs
OD: Optical density
PVP: Polyvinylpyrrolidone
TNF-α: Tumor necrosis factor
TPA: 12-O-Tetradecanoylphorbol-13-acetate
